# Clinical and epidemiological characteristics of pediatric SARS-CoV-2 infections in China: A multicenter case series

**DOI:** 10.1371/journal.pmed.1003130

**Published:** 2020-06-16

**Authors:** Che Zhang, Jiaowei Gu, Quanjing Chen, Na Deng, Jingfeng Li, Li Huang, Xihui Zhou

**Affiliations:** 1 Department of Neonatology, The First Affiliated Hospital of Xi’an Jiaotong University, Xi’an, Shaanxi, People’s Republic of China; 2 Department of Pediatrics, Affiliated Taihe Hospital of Hubei University of Medicine, Shiyan, Hubei, People’s Republic of China; 3 Department of Pediatrics, Dongfeng Hospital of Hubei University of Medicine, Shiyan, Hubei, People’s Republic of China; 4 Pediatric Intensive Care Unit, Shiyan People Hospital, Shiyan, Hubei, People’s Republic of China; London School of Hygiene and Tropical Medicine, UNITED KINGDOM

## Abstract

**Background:**

As of April 18, 2020, over 2,000,000 patients had been diagnosed with coronavirus disease-2019 (COVID-19) globally, and more than 140,000 deaths had been reported. The clinical and epidemiological characteristics of adult patients have been documented recently. However, information on pediatric patients is limited. We describe the clinical and epidemiological characteristics of pediatric patients to provide valuable insight into the early diagnosis and assessment of COVID-19 in children.

**Methods and findings:**

This retrospective, observational study involves a case series performed at 4 hospitals in West China. Thirty-four pediatric patients with COVID-19 were included from January 27 to February 23, 2020. The final follow-up visit was completed by March 16, 2020. Clinical and epidemiological characteristics were analyzed on the basis of demographic data, medical history, laboratory tests, radiological findings, and treatment information. Data analysis was performed for 34 pediatrics patients with COVID-19 aged from 1 to 144 months (median 33.00, interquartile range 10.00–94.25), among whom 14 males (41%) were included. All the patients in the current study presented mild (18%) or moderate (82%) forms of COVID-19. A total of 48% of patients were noted to be without a history of exposure to an identified source. Mixed infections of other respiratory pathogens were reported in 16 patients (47%). Comorbidities were reported in 6 patients (18%). The most common initial symptoms were fever (76%) and cough (62%). Expectoration (21%), vomiting (12%), and diarrhea (12%) were also reported in a considerable portion of cases. A substantial increase was detected in serum amyloid A for 17 patients (among 20 patients with available data; 85%) and in high-sensitivity C-reactive protein for 17 patients (among 29 patients with available data; 59%), whereas a decrease in prealbumin was noticed in 25 patients (among 32 patients with available data; 78%). In addition, significant increases in the levels of lactate dehydrogenase and α-hydroxybutyrate dehydrogenase were detected in 28 patients (among 34 patients with available data; 82%) and 25 patients (among 34 patients with available data; 74%), respectively. Patchy lesions in lobules were detected by chest computed tomographic scans in 28 patients (82%). Ground-glass opacities, which were a typical feature in adults, were rare in pediatric patients (3%). Rapid radiologic progression and a late-onset pattern of lesions in the lobules were also noticed. Lesions in lobules still existed in 24 (among 32 patients with lesions; 75%) patients that were discharged, although the main symptoms disappeared a few days after treatment. All patients were discharged, and the median duration of hospitalization was 10.00 (8.00–14.25) days. The current study was limited by the small sample size and a lack of dynamic detection of inflammatory markers.

**Conclusions:**

Our data systemically presented the clinical and epidemiological features, as well as the outcomes, of pediatric patients with COVID-19. Stratified analysis was performed between mild and moderate cases. The findings offer new insight into early identification and intervention in pediatric patients with COVID-19.

## Abstract 2

Che Zhang and colleagues report on a series of children with SARS-CoV-2 infection in China.

## Author summary

**Why was this study done?:**

- Severe acute respiratory syndrome coronavirus 2 (SARS-CoV-2) infection has spread rapidly worldwide.
- Early identification and intervention are necessary for effective control of the epidemic in both adults and children; however, the information on clinical and epidemiological characteristics in pediatric patients was limited.

**What did the researchers do and find?:**

- We collected and analyzed the clinical data of 34 pediatric patients with coronavirus disease-2019 (COVID-19) in 4 hospitals in China from January 27 to February 23, 2020.
- We described the clinical and epidemiological characteristics of the patients and focused on the features of initial symptoms, radiological findings, and outcomes.
- In contrast to that observed in adult patients, higher proportion of fever, vomiting, and diarrhea were noticed on admission in pediatric cases. Patchy shadows with a high density were common in lobule lesions, whereas the typical features of ground-glass opacities seen in adults were rare in pediatric cases.
- A late-onset pattern of lobule lesions was revealed on the basis of chest computed tomographic scans. The clinical presentations were not as severe as the signs observed in the computed tomography (CT) images, and the recovery of lesions in lobules lagged behind that of the main symptoms.

**What do these findings mean?:**

- The specific clinical features in pediatric patients should be given more attention when physicians are dealing with suspected cases.
- No direct evidence was found in our study regarding the transmission from children to adults. However, the risk of family cluster transmission from children harboring virus and the impact to community-based epidemic prevention should be taken into consideration in policy making for epidemic control.
- With respect to the underlying reasons for the decreased risk of the severe form of COVID-19 in children, mixed infection did not increase protection to ameliorate the disease course of COVID-19 based on our data.

## Introduction

Severe acute respiratory syndrome coronavirus 2 (SARS-CoV-2) infection has spread worldwide rapidly since its emergence in Wuhan in China in early December 2019 [1]. The SARS-CoV-2 epidemic was declared a public health emergency of international concern by the World Health Organization (WHO) on January 30, 2020 [2]. To date, over 2,000,000 patients have been diagnosed with coronavirus disease-2019 (COVID-19) globally. The cumulative number of laboratory-confirmed cases has been reported to be over 660,000 in the United States, 180,000 in Spain, 170,000 in Italy, and 80,000 in China [3]. SARS-CoV-2 was identified as a diverse clade derived from severe acute respiratory syndrome coronavirus (SARS-CoV) and Middle East respiratory syndrome coronavirus (MERS-CoV) and was reported as the cause of COVID-19 [4]. The clinical characteristics of adult patients with COVID-19 have been revealed in recent studies and mainly include fever, cough, dyspnea, and radiographic findings of pneumonia [5–7]. However, information on pediatric patients is limited. This case series describes the clinical and epidemiological features of 34 pediatric patients on the basis of epidemiological, demographic, laboratory, and radiological data and aims to contribute to a comprehensive understanding of the characteristics of COVID-19.

## Methods

### Study design and participants

**Fig 1:**
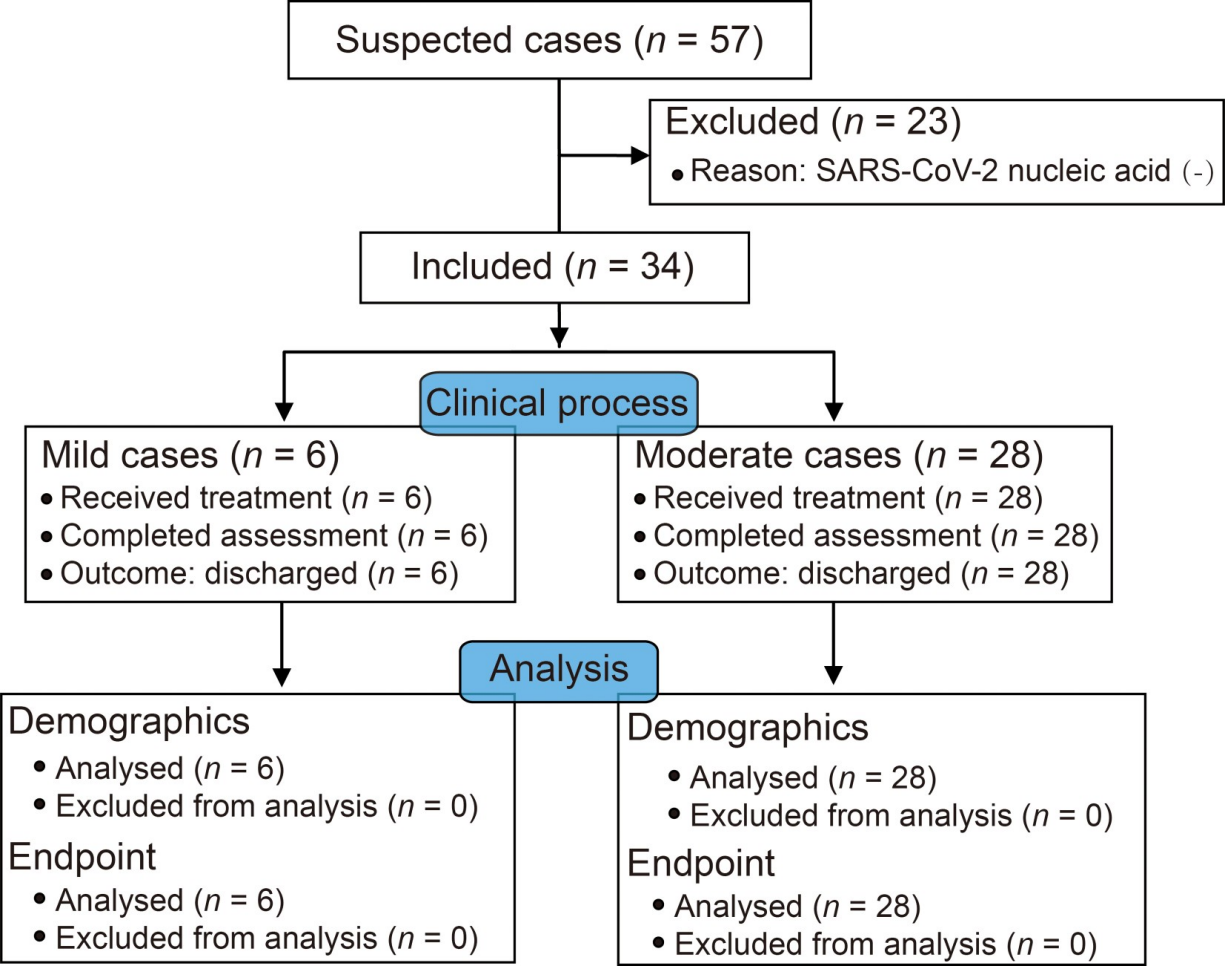
The clinical process and data analysis diagram. SARS-CoV-2, severe acute respiratory syndrome coronavirus 2.

This retrospective, observational study was approved by the institutional review board (IRB) of the Affiliated Taihe Hospital of Hubei University of Medicine (ethical approval no. 2020KY01). Suspected patients with clinical and/or radiological features of pneumonia were quarantined prior to SARS-CoV-2 nucleic acid detection according to WHO guidelines for cases with suspected infection [8] as well as the instructions from the Pediatric Branch of the Hubei Medical Association for pediatric cases [9]. Specifically, suspected cases of SARS-CoV-2 infection should meet 1 of the following criteria [10]: (1) at least 1 clinical symptom, including fever, expectation, tachypnea, lethargy, poor feeding, cough, vomiting, and diarrhea; (2) chest radiologic abnormalities consistent with viral pneumonia. Diagnosis was confirmed by the SARS-CoV-2 nucleic acid test with samples from respiratory tract swabs. Admitted children with laboratory-confirmed SARS-CoV-2-positive results from 4 hospitals in West China from January 27 to February 23, 2020, were included. The clinical type of disease (S1 Table) was assessed for each patient according to the recommendation of the National Health Commission of the People's Republic of China (NHC) [10]. Patients were discharged when all the following criteria were met [10]: (1) fever had recovered for at least 3 days; (2) upper respiratory symptoms were alleviated; (3) the exudative lesion was alleviated significantly according to radiological evidence; (4) negative results were obtained for SARS-CoV-2 nucleic acid detection in 2 consecutive tests performed with an interval of 24 hours. The final follow-up visit was completed by March 16, 2020. Assent was gained from school-aged children, and written informed consent was provided by their parents or guardians prior to data collection. There was no prespecified protocol prior to the current study. The clinical process and data analysis plan are shown in Fig 1.

### Procedures

Samples were taken from nasopharyngeal or throat swabs for SARS-CoV-2 detection. SARS-CoV-2 RNA was detected by real-time reverse transcription polymerase chain reaction (RT-PCR) (S1 Text) in accordance with the recommendation of the NHC [11]. The results of SARS-CoV-2 nucleic acid detection were analyzed by following the manufacturer’s instructions. Cases with negative results were double checked by resampling and retesting with an interval of 24 hours and could be confirmed when negative results were obtained in 2 consecutive tests.

A series of laboratory tests were conducted, including hematological, serum biochemical, acute-phase protein, and erythrocyte sedimentation rate (ESR) testing. In particular, samples from nasopharyngeal or throat swabs were tested for common respiratory pathogens, including influenza A and B virus, respiratory syncytial virus, adenovirus, parainfluenza virus, Epstein–Barr virus, and *Mycoplasma pneumoniae*, using RT-PCR assays with established methods.

Patients underwent chest computed tomography (CT) scans. The radiologic assessments were conducted in Taihe Hospital, which was a treatment center for COVID-19 designated by local municipal government. The images were stored in picture archiving and communication systems (PACS) and reviewed by 2 experienced pediatric radiologists independently. A third radiologist reviewed all CT findings for confirmation.

### Data collection

The medical records of the included patients were accessed by the study team for data collection. Clinical data were extracted, including demographic data, medical history, epidemiological history, underlying diseases, clinical symptoms, signs, laboratory findings, radiological characteristics, treatments, and outcomes. In particular, exposure history was investigated if the patients met any of the following criteria [10]: (1) travel history in Wuhan or neighboring areas or other areas with persistent local transmission within 14 days prior to disease onset; (2) a SARS-CoV-2 infection diagnosis in the child’s family or caregivers; (3) close contact with people who might have or with confirmed SARS-CoV-2 infection or patients with unexplained pneumonia; and (4) children who were associated with a cluster outbreak. In addition, mixed infection was defined as the concurrent infection of a patient with 2 or more pathogens. Two researchers from the Institute of Drug Clinical Trials of Taihe Hospital cross-checked the collected data to ensure quality control and communicated with attending doctors or other healthcare providers if they had any questions. This study was reported based on a STROBE checklist (S1 STROBE Checklist).

### Statistical analysis

Descriptive statistics were determined using SPSS software (version 20.0, IBM, https://www.ibm.com/analytics/spss-statistics-software, Armonk, NY, USA). No imputation was made for missing data. Categorical variables are presented as number and frequency rates. Continuous variables are presented as the median and interquartile range (IQR).

## Results

### Characteristics of the patients

**Fig 2:**
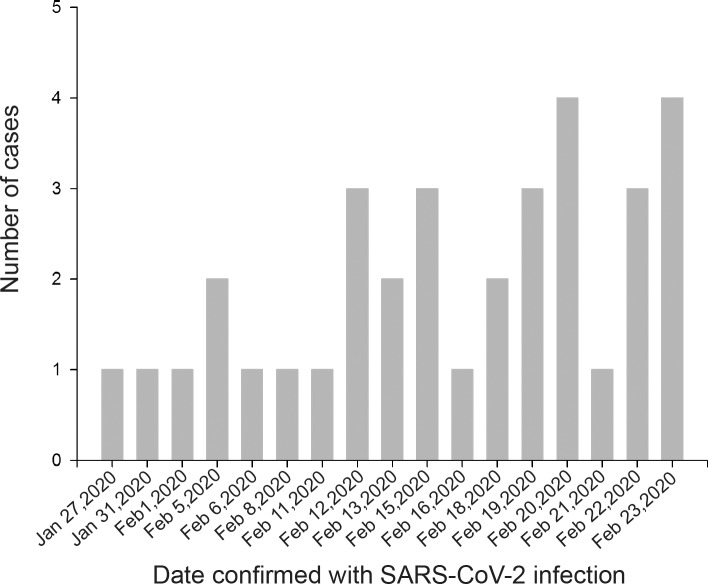
Distribution of cases confirmed with SARS-CoV-2 infection over time. The first patient was diagnosed with SARS-CoV-2 infection on January 27. The daily confirmed cases were increased with time and still went up when the recruitment was cut off on February 23. SARS-CoV-2, severe acute respiratory syndrome coronavirus 2.

In this study, 57 suspected pediatric patients were screened, among whom 34 patients with confirmed COVID-19 were enrolled (Fig 1), including 14 male patients (41%) and 20 female patients (59%). The first patient was diagnosed with SARS-CoV-2 infection on January 27, 9 days after his father was diagnosed with COVID-19. There were 21 cases (62%) that were diagnosed after 15 February, and an uptrend of daily confirmed cases was observed until the cutoff date of our recruitment phase (Fig 2). The median age was 33 (IQR 10.00–94.25) months with a range of 1 to 144 months. Eighteen patients (52%) had exposure to residents of Wuhan. In addition, 13 (38%) patients had close contact with family members with COVID-19, and 16 (48%) patients were noted to be without a history of exposure to an identified source. In particular, mixed infections of other respiratory pathogens were reported in 16 patients (47%), including *M*. *pneumoniae* (26%), influenza B virus (18%), influenza A virus (9%), respiratory syncytial virus (6%), Epstein–Barr virus (6%), parainfluenza virus (3%), and adenovirus (3%). Comorbidities were reported in 6 patients (18%). With respect to the initial symptoms and signs, fever (76%) and cough (62%) were the most frequently complaints. Meanwhile, expectoration (21%), tachypnea (9%), vomiting (12%), and diarrhea (12%) were reported as well. Patients in our study presented mild (18%) or moderate (82%) forms of disease, and moderate cases were predominant (96%) in 23 patients who were not older than 72 months (Table 1).

**Table 1:** Characteristics of patients on admission.

Characteristics	All (*n* = 34)	Age ≤ 12 months	12 < Age ≤ 72 months	Age > 72 months
		(*n* = 10)	(*n* = 13)	(*n* = 11)
Demographics				
Sex				
Male, *n* (%)	14 (41)	6 (60)	3 (23)	5 (45)
Female, *n* (%)	20 (59)	4 (40)	10 (77)	6 (55)
Age, median (IQR), months	33.00 (10.00–94.25)	/	/	/
Clinical type				
Mild, *n* (%)	6 (18)	0	1 (8)	5 (45)
Moderate, *n* (%)	28 (82)	10 (100)	12 (92)	6 (55)
Exposure to suspected cases, *n* (%)	18 (52)	6 (60)	7 (54)	5 (45)
Family cluster, *n* (%)	13 (38)	5 (50)	5 (38)	3 (27)
Mixed infection, *n* (%)	16 (47)	2 (20)	8 (62)	6 (55)
Comorbidities				
Infectious mononucleosis, *n* (%)	2 (6)	/	2 (15)	/
Nephroblastoma, *n* (%)	1 (3)	/	1 (8)	/
Atrial septal defect, *n* (%)	1 (3)	1 (10)	/	/
Febrile convulsion, *n* (%)	1 (3)	/	/	1 (9)
Asthma, *n* (%)	1 (3)	/	/	1 (9)

Abbreviation: IQR, interquartile range

### Laboratory test findings

On admission, hematological tests indicated that the lymphocyte count was increased in 17 patients (50%), although the median value (3.19, 1.73–4.34) was within the normal range. Concerning the findings of blood biochemistry, prealbumin (median 138.65 mg/L) was decreased significantly in 25 patients (among 32 patients with available data; 78%), whereas a substantial increase was detected in serum amyloid A (SAA) for 17 patients (among 20 patients with available data; 85%) and high-sensitivity C-reactive protein (hs-CRP) for 17 patients (among 29 patients with available data; 59%). In addition, a noticeable increase was observed in lactate dehydrogenase (LDH) for 28 patients (among 34 patients; 82%) and in α-hydroxybutyrate dehydrogenase (α-HBDH) for 25 patients (among 34 patients; 74%). However, the results for creatine kinase (CK) and creatine kinase-MB (CK-MB) were normal for all patients (Table 2). No other significant findings were observed in routine blood coagulation tests or urine and stool tests. The results of the electrocardiogram (ECG) exam were normal in all patients during hospitalization. During hospitalization, laboratory tests were reviewed 7 days after admission. The levels of SAA, hs-CRP, and prealbumin had recovered within 7 days posttreatment for all patients with abnormalities at baseline. The median duration for recovery was 7.00 (7.00–10.00) days for LDH and 8.00 (7.00–10.00) days for α-HBDH. The proportion of patients who recovered in LDH and α-HBDH were 86% (for 24 patients) and 84% (for 21 patients), respectively.

**Table 2:** Laboratory findings of patients on admission (*n* = 34).

Laboratory findings	Normal range	Median (IQR)
Hematology		
White blood cell count, ×10^9^ /L	3.50–9.50	6.78 (5.74–8.66)
Neutrophil count, ×10^9^ /L	1.80–6.30	2.96 (2.02–4.34)
Lymphocyte count, ×10^9^ /L	1.10–3.20	3.19 (1.73–4.34)
Platelet count, ×10^9^ /L	150.00–350.00	231.50 (192.75–263.75)
Hemoglobin count, g /L	110.00–160.00	129.00 (116.75–135.00)
Blood biochemistry		
Albumin, g /L	40.00–55.00	44.70 (42.85–46.50)
Prealbumin, mg /L	170.00–420.00	138.65 (106.85–168.55)
ALT, IU /L	0.00–50.00	16.00 (12.75–25.25)
AST, IU /L	0.00–40.00	39.50 (27.00–57.25)
Total bilirubin, μmol /L	3.42–20.50	7.50 (6.10–10.35)
Creatinine, μmol /L	44.00–120.00	42.05 (32.53–49.35)
Potassium, mmol /L	3.50–5.50	4.45 (4.28–4.73)
Sodium, mmol /L	137.00–155.00	139.00 (137.88–141.13)
Alkaline phosphatase, IU /L	0.00–500.00	198.00 (161.00–266.50)
CK, IU /L	50.00–310.00	105.00 (73.75–167.50)
LDH, IU /L	100.00–240.00	327.00 (268.75–403.75)
α-HBDH, IU /L	72.00–182.00	237.00 (179.75–288.00)
CK-MB, IU /L	0.00–24.00	19.00 (14.00–33.50)
Procalcitonin, ng /ml	0.00–0.50	0.06 (0.03–0.07)
SAA, mg /L	0.00–8.00	36.59 (9.25–50.33)
hs-CRP, mg /L	0.00–5.00	7.56 (1.21–15.13)
ESR, mm /h	0.00–20.00	10.00 (8.00–26.00)

Abbreviations: ALT, alanine aminotransferase; AST, aspartate aminotransferase; CK, creatine kinase; CK-MB, creatine kinase-MB; ESR, erythrocyte sedimentation rate; hs-CRP, high-sensitivity C-reactive protein; IQR, interquartile range; LDH, lactate dehydrogenase; SAA, serum amyloid A; α-HBDH, α-hydroxybutyrate dehydrogenase

### Chest CT image findings

**Fig 3:**
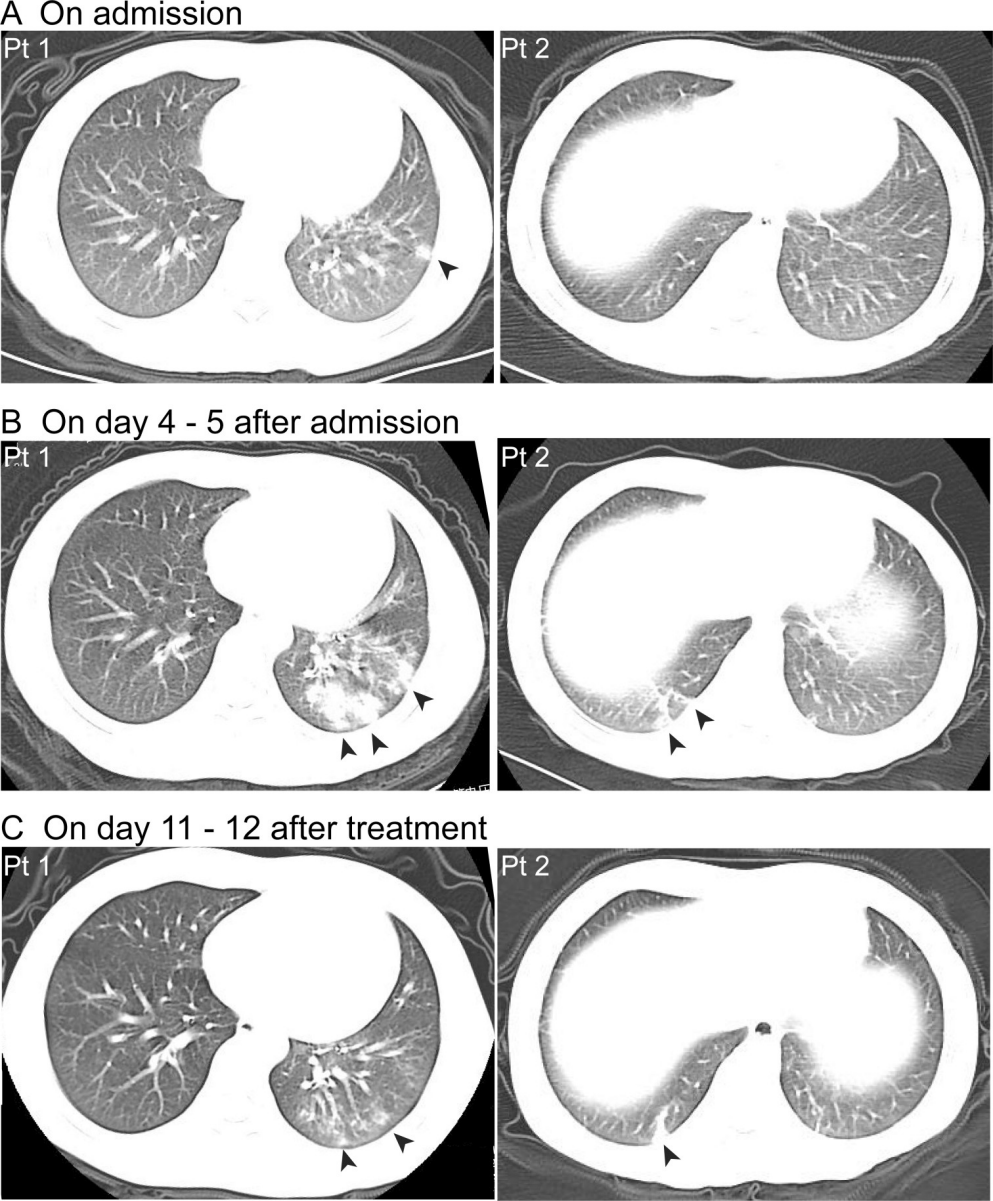
Typical features of CT images of pediatric patients with COVID-19. (A) Patchy shadows of high density in lobule were detected by initial CT images for Pt 1 on admission (on February 1, 2020), whereas no abnormal findings were detected for Pt 2 (on February 5, 2020). (B) Notable progression of lesions was detected for both Pt 1 (on February 6, 2020, day 5 after admission) and Pt 2 (on February 9, 2020, day 4 after admission).(C) The lesions in lobules still existed at discharge for Pt 1 (on February 12, 2020, day 12 after treatment) and Pt 2 (on February 15, 2020, day 11 after treatment). Black arrows point to patchy shadows of high density in lobules. CT, computed tomography; Pt, patient.

Lesions in lobules that were characterized by patchy shadows of high density were indicated by chest CT scans in 28 patients (82%) on admission. A ground-glass opacity with patchy shadows was observed in 1 case (3%) in our study (Table 3). Both unilateral lesions (41%) and bilateral lesions (41%) were detected in the patients with radiological findings. Notable lesion progression was detected in 18 (among 28 patients with lesions on admission; 64%) patients during hospitalization (Patient 1 in Fig 3). Moreover, a late-onset pattern in the chest CT images was observed in 4 cases (among 34 patients; 12%). These patients had normal initial CT images on admission; however, lesions in lobules emerged in 4–5 days thereafter (Patient 2 in Fig 3). There were only 2 cases (among 34 patients; 6%) without emergence of lesions during hospitalization.

**Table 3:** Characteristics and treatments of mild and moderate patients.

Clinical features	All	Mild	Moderate
	(*n* = 34)	(*n* = 6)	(*n* = 28)
Exposure history			
Exposure to suspected cases, *n* (%)	18 (52)	2	16
Unidentified source of infection, *n* (%)	16 (47)	4	12
Mixed infection			
Without mixed infection, *n* (%)	18 (53)	2	16
With mixed infection, *n* (%)	16 (47)	4	12
*M*. *pneumoniae*, *n* (%)	9 (26)	4	5
Influenza B virus, *n* (%)	6 (18)	0	6
Influenza A virus, *n* (%)	3 (9)	0	3
Respiratory syncytial virus, *n* (%)	2 (6)	0	2
Epstein–Barr virus, *n* (%)	2 (6)	1	1
Parainfluenza virus, *n* (%)	1 (3)	0	1
Adenovirus, *n* (%)	1 (3)	0	1
Comorbidities			
With comorbidities, *n* (%)	6 (18)	1	5
Without comorbidities, *n* (%)	28 (82)	5	23
Signs and symptoms			
Fever, *n* (%)	26 (76)	3	23
Cough, *n* (%)	21 (62)	5	16
Expectoration, *n* (%)	7 (21)	0	7
Vomiting, *n* (%)	4 (12)	0	4
Diarrhea, *n* (%)	4 (12)	0	4
Tachypnea, *n* (%)	3 (9)	0	3
CT findings			
Distribution of patchy shadows	28 (82)	0	28
Ground-glass opacity^a^	1 (3)	0	1
Normal, *n* (%)	6 (18)	6	0
Treatments			
Interferon-α nebulization, *n* (%)	34 (100)	6	28
Traditional Chinese medicine, *n* (%)	20 (59)	4	16
Ribavirin, *n* (%)	15 (44)	3	12
Antibiotic therapy, *n* (%)	29 (85)	4	25
Corticosteroid therapy, *n* (%)	5 (15)	0	5
Oxygen inhalation, *n* (%)	3 (9)	0	3

^a^A ground-glass opacity with patchy shadows was observed in 1 case.

Abbreviations: CT, computed tomography

### Treatments and outcomes

Antiviral treatments were employed according to the recommendation of the NHC [10] for mild and moderate cases. All patients received interferon-α nebulization twice a day. Ribavirin was given to 15 (44%) patients twice a day. In addition, 20 (59%) patients received traditional Chinese medicine. Antibiotics were given to 11 patients with an initial diagnosis of bacterial pneumonia on admission before detection of SARS-CoV-2 infection and were withdrawn after confirmation of COVID-19. Nine patients received antibiotic therapy because of concerns about viral-bacterial mixed infections during hospitalization. Azithromycin was given to 9 patients with *M*. *pneumonia* infection. Corticosteroid (15%) and oxygen inhalation supportive therapy (9%) were also employed (Table 3).

**Fig 4:**
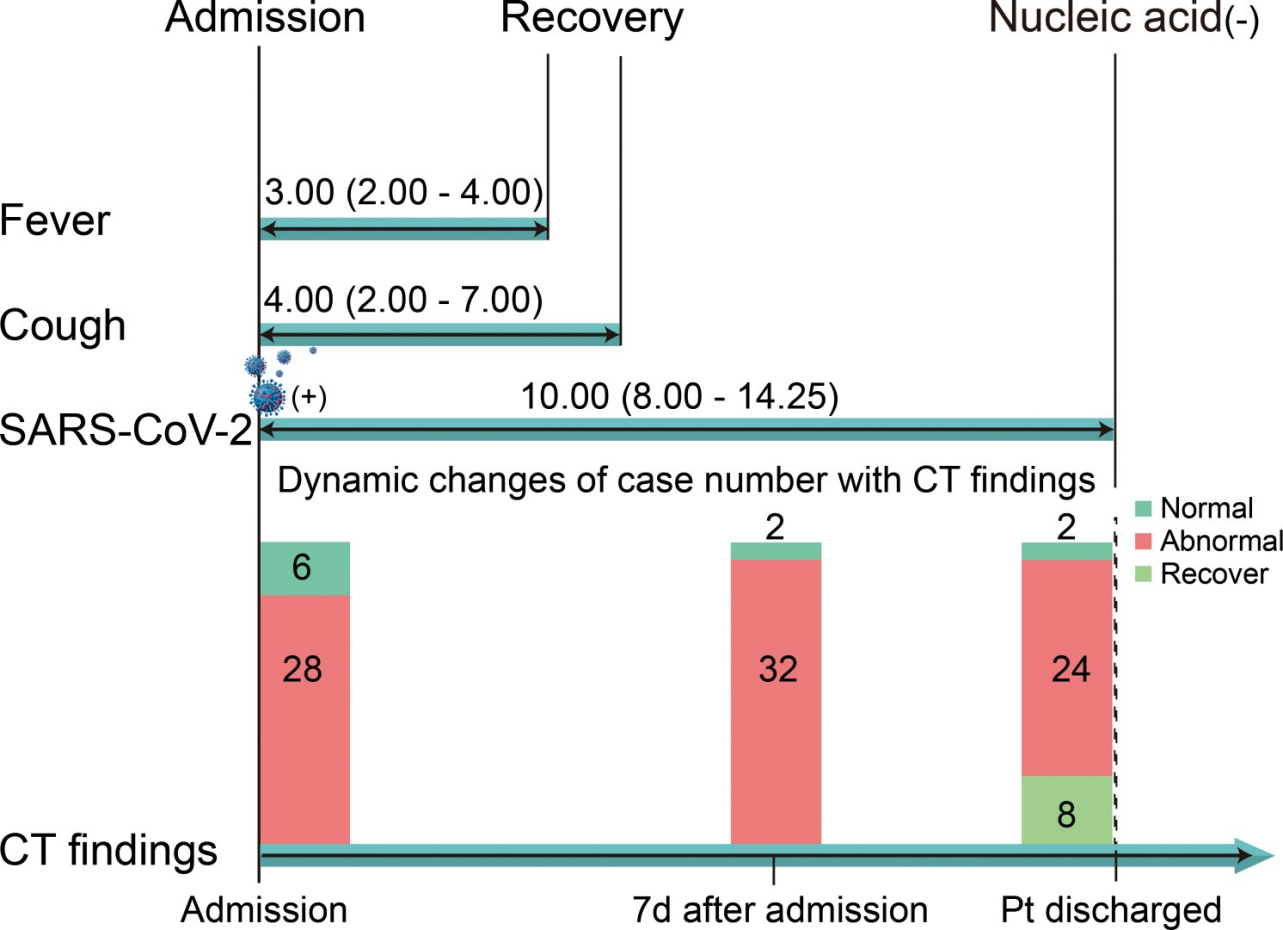
Outcomes of the patients. The duration of the main symptom recovery and the time required for patients to test negative for SARS-CoV-2 RNA are shown as the median (IQR). The dynamic changes in terms of the number of cases among normal, abnormal, and recovered patients are shown to present the progression of radiologic findings. CT, computed tomography; d, days; IQR, interquartile range; Pt, patient; SARS-CoV-2, severe acute respiratory syndrome coronavirus 2.

All patients were discharged once the main symptoms disappeared and the SARS-CoV-2 tests became negative. However, lesions in lobules recovered in only 8 patients. The lesions still existed in 24 patients (among 32 patients with lesions; 75%) when they were discharged (Fig 4). The duration of fever was 3.00 (2.00–4.00) days, similar to that of cough (4.00 days, 2.00–7.00). The duration of hospitalization was 10.00 (8.00–14.25) days for all patients. A shorter duration of hospitalization was indicated in mild cases (8.00 days, 7.00–9.50) than in moderate cases (10.50 days, 8.00–15.00) (Table 4).

**Table 4:** Outcomes of patients.

Outcomes	All	Mild	Moderate
Duration of fever,	3.00	2.00	3.00
median (IQR, *n*), days	(2.00–4.00, 26)	(1.00–3.50, 5)	(2.00–4.00, 21)
Duration of cough,	4.00	4.50	4.00
median (IQR, *n*), days	(2.00–7.00, 21)	(2.50–5.75, 4)	(2.00–7.00, 17)
Duration of hospitalization,	10.00	8.00	10.50
median (IQR, *n*), days^a^	(8.00–14.25, 34)	(7.00–9.50, 6)	(8.00–15.00, 28)
Proportion of patients recovered in lung lesions, *n* (%)^b^	8 (25)	-	8 (25)

^a^Equal to the days when the results of SARS-CoV-2 detection turned to negative.

^b^Data were collected from 32 cases with CT findings when they were discharged.

Abbreviations: CT, computed tomography; IQR, interquartile range; SARS-CoV-2, severe acute respiratory syndrome coronavirus 2

## Discussion

Along with the rapid spread of SARS-CoV-2 infection, the pediatric cases of COVID-19 gradually increased. The morbidity of COVID-19 in children was reported as 0.9% in China [1], 1.2% in Italy [12], and 5% in the USA [13]. However, the clinical and epidemiological characteristics of pediatric patients have not yet been determined clearly. Here, we report the clinical and epidemiological features of 34 pediatric patients with COVID-19 aged from 1 to 144 months. Patients experienced mild or moderate disease forms in the current study. Most patients suffered from fever and cough, which recovered within 3.00–4.00 days after treatment. The progression pattern of the lesions in lobules was revealed by chest CT scan, and the lesions still existed in the majority of patients when discharged. Unlike other reports, the typical feature of ground-glass opacity observed in adults was rare in pediatric patients based on our data. Substantial increases were detected in SSA, hs-CRP, LDH, and α-HDBD, all of which recovered promptly after treatment.

The current study found that all the patients presented mild or moderate COVID-19 disease, which was consistent with the results of previous studies [14,15]. It was also reported that 94% of cases were identified as asymptomatic (4%), mild (51%), or moderate (39%) among 2,143 confirmed and suspected pediatric patients in China [14]. The underlying mechanisms of milder disease presentation in children compared with adults has been a topic of research, and several hypotheses have been raised based on the current understanding of COVID-19. One possible explanation may be related to a reduced inflammatory response due to the less well-developed immune system in children than in adults [16]. However, a substantial increase in hs-CRP was detected in 59% of cases in our study, which was similar to that observed in adult cases (61%) [1]. This finding suggested that an immunological response that was similar to that in adults occurred in the pediatric population in neighboring areas of Wuhan, which did not support the immature immune system theory. Serum inflammatory marker detection was not performed in our study because of the limitation of the retrospective study design, and such detection could be helpful to address this controversial issue in the future. The other theory originated from the observation that younger children experienced milder disease courses. Children of younger age tend to have many viral infections, and it is possible that repeated viral exposure strengthens the immune system when it responds to SARS-CoV-2 [17]. Correspondingly, mixed infection was detected in 16 (47%) patients with other pathogens, including *M*. *pneumoniae*, influenza A and B virus, respiratory syncytial virus, Epstein–Barr virus, parainfluenza virus, and adenovirus. However, all these pathogens were tested to be negative in 10 pediatric cases from Guangzhou [18]. Stratified analysis according to age range was performed to determine the correlation between age and mixed infection, if any, as well as the impact of mixed infection on the clinical type of disease. As a result, mixed infection (62% in 13 patients) was most common in children aged between 12 and 72 months, and 12 moderate cases (92% in 13 patients) were identified in this subgroup. It was suggested that mixed infection did not increase protection to ameliorate the disease course of COVID-19 based on our data. In addition, children with moderate disease aged below 72 months accounted for 79% of all moderate cases, suggesting that preschool children were more prone to developing SARS-CoV-2 infection.

According to our current data from the chest CT images, patchy shadows were detected in 82% of patients on admission, which was in accordance with previous reports in adults (86%) [1] and children (65%) [15]. Lesions in lobules were characterized with patchy shadows of high density in most cases (97%). Ground-glass opacity was rare (3%) in the current study, although it was common in pediatric cases from Wuhan (33% in 171 cases) [15] and Guangzhou (50% in 10 cases) [18], as well as in adults (56%) [19]. Notably, the proportion of patients with a history of exposure was 52% in current study, whereas the proportion was 90% in 171 cases from Wuhan [15] and 100% in 10 cases from Guangzhou [18]. Thus, exposure status might attribute partially to the discrepancy of proportions in pediatric cases between current study and previous studies. Further study was needed to reveal the correlativity between viral load of SARS-CoV-2 and exposure status to identify the underlying reason for the discrepancy. The time course of lung changes was revealed in adult patients [20]; however, the course of progression remained elusive in pediatric cases. Notably, severe progression of lesions in lobules was noticed within 7 days after admission in the current study and even sometimes appeared 4–5 days after admission. However, the clinical presentations were not so severe as the signs shown in CT images. Rapid radiologic progression was also reported, with a peak at approximately 2 weeks after onset [21] in adult cases. In addition, a late-onset pattern of lesions was detected in some cases, because the lesions were indicated by the CT scan after approximately 7 days after symptom onset, which was similar to that observed in other report for adults (6–12 days) [22]. Nonetheless, our findings suggest that close monitoring for pediatric patients should be performed because of the severe progression of lesions in lobules and the late-onset pattern seen in some cases.

The level of SAA was found to be increase in a high percentage (for 17 patients among 20 patients with available data; 85%) of patients undergoing the test and was a sensitive marker correlated with the extent of pneumonia in SARS patients [23]. The levels of hs-CRP and SAA recovered dramatically within 7 days after treatments. The correlation of SAA and SARS-CoV-2 infection remains to be investigated in pediatric patients. Consistent with previous reports in adults [1,6] and children [24], the levels of LDH and α-HBDH were increased without any symptoms or signs of myocardial impairment.

With respect to the initial symptoms, fever was identified in 26 children (76%) in our study; however, it presents in only 44% of adult patients on admission [1]. In addition, vomiting (12%) and diarrhea (12%) also present on admission and were more common in children than in adult patients (5% for vomiting and 4% for diarrhea) [1]. Comorbidities were found in 6 patients (18%) in the current study, which was similar to that observed in adult patients with mild symptoms (21%) [1].

The therapeutic strategy was based on antiviral therapies, which was in alignment with the recommendations of the NHC [10]. All the patients had recovered from the main symptoms when discharged. A negative SARS-CoV-2 detection result was achieved in 10.00 (8.00–14.25) days. Lesions in lobules still existed in 75% of patients, although great improvements were shown in CT scans after treatments. An association of the radiologic findings with mortality was revealed in adult patients [19]. However, it was not suggested to utilize CT scans for prognosis prediction in mild and moderate cases because no definitive correlation was found between radiologic imaging and the course of the disease in our study.

Our study also adds new information to existing reports on epidemiological characteristics. A considerable percentage of pediatric patients (48%) was noticed to have an unidentified source of infection, whereas up to 72% of nonresidents of Wuhan had contact with residents of Wuhan [1]. The unanticipated findings suggested that the reference value of exposure history to epidemic areas for the early identification of SARS-CoV-2 infection should be considered carefully for pediatric patients during the rapid development of epidemics. The correlation of exposure history with disease severity could be investigated in a future study with a larger population. In accordance with the present studies [14,25], family cluster transmission was found to be common in our pediatric patients. There have been few reports of the infection dynamics from pediatric patients to their caregivers, although transmission from adults to children has been identified with confirmed evidence. Children may become potential spreaders in the explosive stage of the outbreak, which was attributed to a high prevalence of asymptomatic infection and milder disease in the pediatric population [25]. Thus, a close monitoring and tracking system involving hospitals and communities was utilized to track the transmission between pediatric patients and their caregivers. However, no evidence was shown regarding the transmission route from pediatric patients to their caregivers and close-contact family members.

The patient population in the current study is representative of pediatric cases diagnosed and treated in West China. However, the interpretation of our findings was limited by the small sample size and retrospective study design. The underlying reasons for the lower risk of the severe form of COVID-19 in children remain elusive because of a lack of dynamic detection of the viral load of SARS-CoV-2 and inflammatory markers. Further information about these issues would help us to obtain a broader view of COVID-19.

### Conclusion

This case series described the clinical and epidemiological characteristics of pediatric patients with COVID-19. Our data presented the clinical features of pediatric patients to facilitate early identification and intervention in suspected patients. Notwithstanding the relatively limited number of samples, our findings offer valuable insight into the early diagnosis and epidemic control of COVID-19 in children.

## Supporting information

S1 STROBE Checklist Reporting items of observational studies. (DOCX)

S1 Table Clinical type of COVID-19. COVID-19, coronavirus disease-2019.
(DOCX)

S1 Text Process of RT-PCR for SARS-CoV-2 detection. RT-PCR, real-time reverse transcription polymerase chain reaction; SARS-CoV-2, severe acute respiratory syndrome coronavirus 2.
(DOCX)

## Abbreviations

ALT: alanine aminotransferase
AST: aspartate aminotransferase
CK: creatine kinase
CK-MB: creatine kinase-MB
COVID-19: coronavirus disease-2019
CT: computed tomography
ECG: electrocardiogram
ESR: erythrocyte sedimentation rate
hs-CRP: high-sensitivity C-reactive protein
IQR: interquartile range
IRB: institutional review board
LDH: lactate dehydrogenase
MERS-CoV: Middle East respiratory syndrome coronavirus
NHC: National Health Commission of the People’s Republic of China
PACS: picture archiving and communication systems
RT-PCR: real-time reverse transcription polymerase chain reaction
SAA: serum amyloid A
SARS-CoV: severe acute respiratory syndrome coronavirus
SARS-CoV-2: severe acute respiratory syndrome coronavirus 2
WHO: World Health Organization
α-HBDH: α-hydroxybutyrate dehydrogenase
