# Supplementary material for: Clinical and epidemiological characteristics of pediatric SARS-CoV-2 infections in China: A multicenter case series
Source: PLoS Med. 2020 Jun 16;17(6):e1003130. doi: 10.1371/journal.pmed.1003130 (PMC7297312; doi:10.1371/journal.pmed.1003130)
Supplement: S1 STROBE Checklist — (DOCX) [file pmed.1003130.s001.docx]

S1 STROBE Checklist.

STROBE Statement—checklist of items that should be included in reports of observational studies

|  | Item No | Recommendation | Section, Paragraph No. | | Relevant text from manuscript |
| --- | --- | --- | --- | --- | --- |
| **Title and abstract** | 1 | (*a*) Indicate the study’s design with a commonly used term in the title or the abstract | Title | | Clinical and Epidemiological Characteristics of Pediatric SARS-CoV-2 Infections in China: a Multicenter Case Series |
|  |  | (*b*) Provide in the abstract an informative and balanced summary of what was done and what was found | Abstract,  Para 3 | | Our data systemically presented the clinical and epidemiological features, as well as the outcomes, of pediatric patients with COVID-19. Stratified analysis was performed between mild and moderate cases. The findings offer new insight into early identification and intervention in pediatric patients with COVID-19. |
|  | | | | Introduction | |
| Background/rationale | 2 | Explain the scientific background and rationale for the investigation being reported | Para 1 | | The clinical characteristics of adult patients with COVID-19 have been revealed in recent studies and mainly include fever, cough, dyspnea, and radiographic findings of pneumonia. However, information on pediatric patients is limited. |
| Objectives | 3 | State specific objectives, including any prespecified hypotheses | Para 1 | | This case series describes the clinical and epidemiological features of 34 pediatric patients on the basis of epidemiological, demographic, laboratory and radiological data, and aims to contribute to a comprehensive understanding of the characteristics of COVID-19. |
|  | | | Methods | | |
| Study design | 4 | Present key elements of study design early in the paper | Para 1  (Study Design and Participants) | | This retrospective, observational study was approved by the institutional review board (IRB) of the Affiliated Taihe Hospital of Hubei University of Medicine (ethical approval no.2020KY01). |
| Setting | 5 | Describe the setting, locations, and relevant dates, including periods of recruitment, exposure, follow-up, and data collection | Para 1  (Study Design and Participants) | | Admitted children with laboratory-confirmed SARS-CoV-2-positive results from 4 hospitals in West China from January 27 to February 23, 2020 were included. |
| Participants | 6 | (*a*) *Cohort study*—Give the eligibility criteria, and the sources and methods of selection of participants. Describe methods of follow-up  *Case-control study*—Give the eligibility criteria, and the sources and methods of case ascertainment and control selection. Give the rationale for the choice of cases and controls  *Cross-sectional study*—Give the eligibility criteria, and the sources and methods of selection of participants | Para 1  (Study Design and Participants) | | Admitted children with laboratory-confirmed SARS-CoV-2-positive results from 4 hospitals in West China from January 27 to February 23, 2020 were included. |
|  |  | (*b*) *Cohort study*—For matched studies, give matching criteria and number of exposed and unexposed  *Case-control study*—For matched studies, give matching criteria and the number of controls per case |  | | Not applicable. |
| Variables | 7 | Clearly define all outcomes, exposures, predictors, potential confounders, and effect modifiers. Give diagnostic criteria, if applicable | Para 1  (Study Design and Participants) | | Specifically, suspected cases of SARS-CoV-2 infection should meet one of the following criteria [10] : (a) at least one clinical symptom, including fever, expectation, tachypnea, lethargy, poor feeding, cough, vomiting, and diarrhea; (b) chest radiologic abnormalities consistent with viral pneumonia. Diagnosis was confirmed by the SARS-CoV-2 nucleic acid test with samples from respiratory tract swabs. |
| Data sources/ measurement | 8* | For each variable of interest, give sources of data and details of methods of assessment (measurement). Describe comparability of assessment methods if there is more than one group | Para 5  (Data Collection) | | The medical records of the included patients were accessed by the study team for data collection. Clinical data were extracted, including demographic data, medical history, epidemiological history, underlying diseases, clinical symptoms, signs, laboratory findings, radiological characteristics, treatments and outcomes. In particular, exposure history was investigated if the patients met any of the following criteria: (a) travel history in Wuhan or neighbouring areas or other areas with persistent local transmission within 14 days prior to disease onset; (b) a SARS-CoV-2 infection diagnosis in the child’s family or caregivers; (c) close contact with people who might have or with confirmed SARS-CoV-2 infection or patients with unexplained pneumonia; and (d) children who were associated with a cluster outbreak. In addition, mixed infection was defined as the concurrent infection of a patient with two or more pathogens. |
| Bias | 9 | Describe any efforts to address potential sources of bias | Para 5  (Data Collection) | | Two researchers from the Institute of Drug Clinical Trials of Taihe Hospital cross-checked the collected data to ensure quality control and communicated with attending doctors or other healthcare providers if they had any questions. |
| Study size | 10 | Explain how the study size was arrived at | Para 1  (Study Design and Participants) | | Admitted children with laboratory-confirmed SARS-CoV-2-positive results from 4 hospitals in West China from January 27 to February 23, 2020 were included. |
| Quantitative variables | 11 | Explain how quantitative variables were handled in the analyses. If applicable, describe which groupings were chosen and why | Para 6  (Statistical Analysis) | | Continuous variables are presented as the median and interquartile range (IQR). |
| Statistical methods | 12 | (*a*) Describe all statistical methods, including those used to control for confounding | Para 6  (Statistical Analysis) | | Descriptive statistics were determined using SPSS software (version 20.0, IBM, Armonk, NY, USA). No imputation was made for missing data. Categorical variables are presented as number and frequency rates. Continuous variables are presented as the median and interquartile range (IQR). |
|  |  | (*b*) Describe any methods used to examine subgroups and interactions |  | | Not applicable. |
|  |  | (*c*) Explain how missing data were addressed | Para 6  (Statistical Analysis) | | No imputation was made for missing data. |
|  |  | (*d*) *Cohort study*—If applicable, explain how loss to follow-up was addressed  *Case-control study*—If applicable, explain how matching of cases and controls was addressed  *Cross-sectional study*—If applicable, describe analytical methods taking account of sampling strategy |  | | Not applicable. |
|  |  | (*e*) Describe any sensitivity analyses |  | | Not applicable. |

|  | | Results | | | |
| --- | --- | --- | --- | --- | --- |
| Participants | 13* | (a) Report numbers of individuals at each stage of study—eg numbers potentially eligible, examined for eligibility, confirmed eligible, included in the study, completing follow-up, and analysed | Para 1  (Characteristics of the Patients) | In this study, 57 suspected pediatric patients were screened, among whom 34 patients with confirmed COVID-19 were enrolled (Fig 1), including 14 male patients (42%) and 20 female patients (58%). | |
|  |  | (b) Give reasons for non-participation at each stage | Figure 1 | Fig 1. The Clinical Process and Data Analysis Diagram. | |
|  |  | (c) Consider use of a flow diagram | Figure 1 | Fig 1. The Clinical Process and Data Analysis Diagram. | |
| Descriptive data | 14* | (a) Give characteristics of study participants (eg demographic, clinical, social) and information on exposures and potential confounders | Para 1  (Characteristics of the Patients) | In this study, 57 suspected pediatric patients were screened, among whom 34 patients with confirmed COVID-19 were enrolled (Fig 1), including 14 male patients (41%) and 20 female patients (59%). The first patient was diagnosed with SARS-CoV-2 infection at January 27, 9 days after his father was diagnosed with COVID-19. There were 21 cases (62%) were diagnosed after 15 February, and an uptrend of daily confirmed cases was observed until the cut-off date of our recruitment phase (Fig 2). The median age was 33 (IQR 10.00 - 94.25) months with a range of 1 to 144 months. Eighteen patients (52%) had exposure to residents of Wuhan. In addition, 13 (38%) patients had close contact with family members with COVID-19, and 16 (48%) patients were noted to be without a history of exposure to an identified source. In particular, mixed infections of other respiratory pathogens were reported in 16 patients (47%), including Mycoplasma pneumoniae (26%), influenza B virus (18%), influenza A virus (9%), respiratory syncytial virus (6%), Epstein-Barr virus (6%), parainfluenza virus (3%), and adenovirus (3%). Comorbidities were reported in 6 patients (18%). With respect to the initial symptoms and signs, fever (76%) and cough (62%) were the most frequently complaints. Meanwhile, expectoration (21%), tachypnea (9%), vomiting (12%), and diarrhea (12%) were reported as well. Patients in our study presented mild (18%) or moderate (82%) forms of disease, and moderate cases were predominant (96%) in 23 patients who were not older than 72 m (Table 1). | |
|  |  | (b) Indicate number of participants with missing data for each variable of interest |  | Not applicable. | |
|  |  | (c) *Cohort study*—Summarise follow-up time (eg, average and total amount) |  | Not applicable. | |
| Outcome data | 15* | *Cohort study*—Report numbers of outcome events or summary measures over time |  |  | |
|  |  | *Case-control study—*Report numbers in each exposure category, or summary measures of exposure |  |  | |
|  |  | *Cross-sectional study—*Report numbers of outcome events or summary measures | Para 4  (Treatments and Outcomes) | Antiviral treatments were employed according to the recommendation of the NHC [10] for mild and moderate cases. All patients received interferon-α nebulization twice a day. Ribavirin was given to 15 (44%) patients twice a day. In addition, 20 (59%) patients received traditional Chinese medicine. Antibiotics were given to 11 patients with an initial diagnosis of bacterial pneumonia on admission before detection of SARS-CoV-2 infection and were withdrawn after confirmation of COVID-19. Nine patients received antibiotic therapy due to concerns about viral-bacterial mixed infections during hospitalization. Azithromycin was given to 9 patients with *Mycoplasma pneumonia* infection. Corticosteroid (15%) and oxygen inhalation supportive therapy (9%) were also employed (Table 3).  All patients were discharged once the main symptoms disappeared and the SARS-CoV-2 tests became negative. However, lesions in lobules recovered in only 8 patients. The lesions still existed in 24 patients (75%) when they were discharged (Fig 4). The duration of fever was 3.00 (2.00 - 4.00) days, similar to that of cough (4.00 days, 2.00 - 7.00). The duration of hospitalization was 10.00 (8.00 - 14.25) days for all patients. A shorter duration of hospitalization was indicated in mild cases (8.00 days, 7.00 - 9.50) than in moderate cases (10.50 days, 8.00 - 15.00) (Table 4). | |
| Main results | 16 | (*a*) Give unadjusted estimates and, if applicable, confounder-adjusted estimates and their precision (eg, 95% confidence interval). Make clear which confounders were adjusted for and why they were included |  | Not applicable. | |
|  |  | (*b*) Report category boundaries when continuous variables were categorized | Para 1  (Characteristics of the Patients) | Table 1 | |
|  |  | (*c*) If relevant, consider translating estimates of relative risk into absolute risk for a meaningful time period |  | Not applicable. | |
| Other analyses | 17 | Report other analyses done—eg analyses of subgroups and interactions, and sensitivity analyses |  | Not applicable. | |
|  | | Discussion | | | |
| Key results | 18 | Summarise key results with reference to study objectives | Para 1 | Along with the rapid spread of SARS-CoV-2 infection, the pediatric cases of COVID-19 gradually increased. The morbidity of COVID-19 in children was reported as 0.9% in China [1], 1.2% in Italy [12], and 5% in the USA [13]. However, the clinical and epidemiological characteristics of pediatric patients have not yet been determined clearly. Here, we report the clinical and epidemiological features of 34 pediatric patients with COVID-19 aged from 1 to 144 months. Patients experienced mild or moderate disease forms in the current study. Most patients suffered from fever and cough, which recovered within 3.00 - 4.00 days after treatment. The progression pattern of the lesions in lobules was revealed by chest CT scan, and the lesions still existed in the majority of patients when discharged. Unlike other reports, the typical feature of ground-glass opacity observed in adults was rare in pediatric patients based on our data. Substantial increases were detected in SSA, hs-CRP, LDH, and α- HDBD, all of which recovered promptly after treatment. | |
| Limitations | 19 | Discuss limitations of the study, taking into account sources of potential bias or imprecision. Discuss both direction and magnitude of any potential bias | Para 8 | The patient population in the current study is representative of pediatric cases diagnosed and treated in West China. However, the interpretation of our findings was limited by the small sample size and retrospective study design. The underlying reasons for the lower risk of the severe form of COVID-19 in children remain elusive due to a lack of dynamic detection of the viral load of SARS-CoV-2 and inflammatory markers. | |
| Interpretation | 20 | Give a cautious overall interpretation of results considering objectives, limitations, multiplicity of analyses, results from similar studies, and other relevant evidence | Para 2 ~ 7 | The current study found that all the patients presented mild or moderate COVID-19 disease, which was consistent with the results of previous studies [14, 15]. It was also reported that 94% of cases were identified as asymptomatic (4%), mild (51%), or moderate (39%) among 2143 confirmed and suspected pediatric patients in China [14]. The underlying mechanisms of milder disease presentation in children compared to adults has been a topic of research, and several hypotheses have been raised based on the current understanding of COVID-19. One possible explanation may be related to a reduced inflammatory response due to the less well-developed immune system in children than in adults [16]. However, a substantial increase in hs-CRP was detected in 59% of cases in our study, which was similar to that observed in adult cases (61%) [1]. This finding suggested that an immunological response that was similar to that in adults occurred in the pediatric population in neighbouring areas of Wuhan, which did not support the immature immune system theory. Serum inflammatory marker detection was not performed in our study due to the limitation of the retrospective study design, and such detection could be helpful to address this controversial issue in the future. The other theory originated from the observation that younger children experienced milder disease courses. Children of younger age tend to have many viral infections, and it is possible that repeated viral exposure strengthens the immune system when it responds to SARS-CoV-2 [17]. Correspondingly, mixed infection was detected in 16 (47%) patients with other pathogens, including Mycoplasma pneumoniae, influenza A and B virus, respiratory syncytial virus, epstein-barr virus, parainfluenza virus, and adenovirus. However, all these pathogens were tested to be negative in 10 pediatric cases from Guangzhou [18]. Stratified analysis according to age range was performed to determine the correlation between age and mixed infection, if any, as well as the impact of mixed infection on the clinical type of disease. As a result, mixed infection (62% in 13 patients) was most common in children aged between 12 and 72 m, and 12 moderate cases (92% in 13 patients) were identified in this subgroup. It was suggested that mixed infection did not increase protection to ameliorate the disease course of COVID-19 based on our data. In addition, children with moderate disease aged below 72 m accounted for 79% of all moderate cases, suggesting that preschool children were more prone to developing SARS-CoV-2 infection.  According to our current data from the chest CT images, patchy shadows were detected in 82% of patients on admission, which was in accordance with previous reports in adults (86%) [1] and children (65%) [15]. Lesions in lobules were characterized with patchy shadows of high density in most cases (97%). Ground-glass opacity was rare (3%) in current study, although it was common in pediatric cases from Wuhan (33% in 171 cases) [15] and Guangzhou (50% in 10 cases) [18], as well as in adults (56%) [19]. Notably, the proportion of patients with a history of exposure was 52% in current study, while the proportion was 90% in 171 cases from Wuhan [15] and 100% in 10 cases from Guangzhou [18]. Thus, exposure status might attribute partially to the discrepancy of proportions in pediatric cases between current study and previous studies. Further study was needed to reveal the correlativity between viral load of SARS-CoV-2 and exposure status to identify the underlying reason for the discrepancy. The time course of lung changes was revealed in adult patients [20]; however, the course of progression remained elusive in pediatric cases. Notably, aggressive development of lesions in lobules was noticed within 7 days after admission in the current study, and even sometimes appeared 4 - 5 days after admission. However, the clinical presentations were not so aggressive as the signs shown in CT images. Rapid radiologic progression was also reported, with a peak at approximately 2 weeks after onset [21] in adult cases. In addition, a late-onset pattern of lesions was detected in some cases, since the lesions were indicated by the CT scan after approximately 7 days after symptom onset, which was similar to that observed in other report for adults (6 - 12 days) [22]. Nonetheless, our findings suggest that close monitoring for pediatric patients should be performed due to the aggressive development of lesions in lobules, and the late-onset pattern seen in some cases.  The level of SAA was found to be increase in a high percentage (85%) of patients undergoing the test and was a sensitive marker correlated with the extent of pneumonia in SARS patients [23]. The levels of hs-CRP and SAA recovered dramatically within 7 days after treatments. The correlation of SAA and SARS-CoV-2 infection remains to be investigated in pediatric patients. Consistent with previous reports in adults [1, 6] and children [24], the levels of LDH and α-HBDH were increased without any symptoms or signs of myocardial impairment.  With respect to the initial symptoms, fever was identified in 26 children (76%) in our study; however, it present in only 44% of adult patients on admission [1]. In addition, vomiting (12%) and diarrhea (12%) also present on admission and were more common in children than in adult patients (5% for vomiting and 4% for diarrhea) [1]. Comorbidities were found in 6 patients (18%) in the current study, which was similar to that observed in adult patients with mild symptoms (21%) [1]. The therapeutic strategy was based on antiviral therapies, which was in alignment with the recommendations of the NHC [10]. All the patients had recovered from the main symptoms when discharged. A negative SARS-CoV-2 detection result was achieved in 10.00 (8.00 - 14.25) days. Lesions in lobules still existed in 75% of patients, although great improvements were shown in CT scans after treatments. An association of the radiologic findings with mortality was revealed in adult patients [19]. However, it was not suggested to utilize CT scans for prognosis prediction in mild and moderate cases since no definitive correlation was found between radiologic imaging and the course of the disease in our study.  Our study also inputted additional information from previous reports on epidemiological characteristics. A considerable percentage of pediatric patients (48%) was noticed to have an unidentified source of infection, while up to 72% of nonresidents of Wuhan had contact with residents of Wuhan [1]. The unanticipated findings suggested that the reference value of exposure history to epidemic areas for the early identification of SARS-CoV-2 infection should be considered carefully for pediatric patients during the rapid development of epidemics. The correlation of exposure history with disease severity could be investigated in a future study with a larger population. In accordance with the present studies [14, 25], family cluster transmission was found to be common in our pediatric patients. There have been few reports of the infection dynamics from pediatric patients to their caregivers, although transmission from adults to children has been identified with confirmed evidence. Children may become potential spreaders in the explosive stage of the outbreak, which was attributed to a high prevalence of asymptomatic infection and milder disease in the pediatric population [25]. Thus, a close monitoring and tracking system involving hospitals and communities were utilized to track the transmission between pediatric patients and their caregivers. However, no evidence was shown regarding the transmission route from pediatric patients to their caregivers and close-contact family members. | |
| Generalisability | 21 | Discuss the generalisability (external validity) of the study results | Para 9  (Conclusion) | This case series described the clinical and epidemiological characteristics of pediatric patients with COVID-19. Our data presented the clinical features of pediatric patients to facilitate early identification and intervention in suspected patients. Notwithstanding the relatively limited number of samples, our findings offer valuable insight into the early diagnosis and epidemic control of COVID-19 in children. | |
|  | | Other information | | | |
| Funding | 22 | Give the source of funding and the role of the funders for the present study and, if applicable, for the original study on which the present article is based | | Not applicable. |  |

*Give information separately for cases and controls in case-control studies and, if applicable, for exposed and unexposed groups in cohort and cross-sectional studies.

**Note:** An Explanation and Elaboration article discusses each checklist item and gives methodological background and published examples of transparent reporting. The STROBE checklist is best used in conjunction with this article (freely available on the Web sites of PLoS Medicine at http://www.plosmedicine.org/, Annals of Internal Medicine at http://www.annals.org/, and Epidemiology at http://www.epidem.com/). Information on the STROBE Initiative is available at www.strobe-statement.org.
