# Supplementary material for: Clinical and epidemiological characteristics of pediatric SARS-CoV-2 infections in China: A multicenter case series
Source: PLoS Med. 2020 Jun 16;17(6):e1003130. doi: 10.1371/journal.pmed.1003130 (PMC7297312; doi:10.1371/journal.pmed.1003130)
Supplement: S1 Table — COVID-19, coronavirus disease-2019. (DOCX) [file pmed.1003130.s002.docx]

| Clinical type | Mild disease | Moderate disease | Severe disease | Critical illness |
| --- | --- | --- | --- | --- |
| Positive RT-PCR test for SARS-CoV-2 | **+** | **+** | **+** | **+** |
| Upper respiratory symptoms | **+/-** asymptomatic infection | **+** | **+** | **+** |
| Mild pneumonia | **-** | **+** | **+** | **+** |
| Abnormal radiographic presentation | **-** | **+** | **+** | **+** |
| Any of manifestations that suggest disease progression ^a^ | **-** | **-** | **+** | **+** Rapid disease progression |
| Any critical conditions ^b^ | **-** | **-** | **-** | **+** |

S 1 Table. Clinical Type of COVID-19.

^a^ Manifestations that suggest disease progression:

• Rapid breath (≥70 breaths per min for infants aged <1 year; ≥50 breaths per min for children aged >1 year)

• Hypoxia

• Lack of consciousness, depression, coma, convulsions

• Dehydration, difficulty feeding, gastrointestinal dysfunction

• Myocardial injury

• Elevated liver enzymes

• Coagulation dysfunction, rhabdomyolysis, and any other manifestations suggesting injuries to vital organs

^b^ Critical conditions

• Respiratory failure with need for mechanical ventilation (eg, ARDS, persistent hypoxia that cannot be alleviated by inhalation through nasal catheters or masks)

• Septic shock

• Organ failure that needs monitoring in the ICU
