# Supplementary material for: Clinical and epidemiological characteristics of pediatric SARS-CoV-2 infections in China: A multicenter case series
Source: PLoS Med. 2020 Jun 16;17(6):e1003130. doi: 10.1371/journal.pmed.1003130 (PMC7297312; doi:10.1371/journal.pmed.1003130)
Supplement: S1 Text — RT-PCR, real-time reverse transcription polymerase chain reaction; SARS-CoV-2, severe acute respiratory syndrome coronavirus 2. (DOCX) [file pmed.1003130.s003.docx]

S 1 Text. Process of RT-PCR for SARS-CoV-2 Detection.

RNA was extracted from samples using RNA isolation kit (SDK60103, bioPerfectus technologies, Taizhou, China) according to the manufacturer’s instructions. After lysis and centrifugation, the suspension was collected for SARS-CoV-2 nucleic acid detection. RT-PCR assay was preformed using SARS-CoV-2 specific kit (NC-ORF1ab/N, DAAN GENE, Guangzhou, China). The adapter primers and probe were supplied with kits, targeting to the open reading frame 1ab (*ORF1ab*) and nucleocapsid protein (*N*) of CoV gene. Specifically, for target *ORF1ab*: forward primer sequence CCCTGTGGGTTTTACACTTAA; reverse primer sequence ACGATTGTGCATCAGCTGA; and the probe sequence 5'-FAM-CCGTCTGCGGTATGTGGAAAGGTTATGG-BHQ1-3'. For target *N*: forward primer sequence GGGGAACTTCTCCTGCTAGAAT; reverse primer sequence CAGACATTTTGCTCTCAAGCTG; and the probe sequence 5'-FAM-TTGCTGCTGCTTGACAGATT-TAMRA-3'. Amplifications were performed according to manufacturer’s protocol under following conditions: reverse transcription at 50°C for 15 min, and Taq inhibitor inactivation at 95°C for 15 min, followed by 45 cycles of denaturation at 94°C for 15 s, and extension at 55°C for 45 s. The results of SARS-CoV-2 nucleic acid detection were analyzed following the manufacturer’s instructions. A test result with a cycle threshold value (Ct-value) ≤ 38 and a significant amplification curve was defined as positive. A negative result was defined with a Ct-value > 38. Retesting was required in cases other than those mentioned above.
